# Isolation and Antioxidant Mechanism of Polyphenols from *Sanghuangporous vaninii*

**DOI:** 10.3390/antiox13121487

**Published:** 2024-12-05

**Authors:** Peng Liu, Yuyang Wang, Daoyou Chen, Zhengpeng Li, Di Wu, Zhong Zhang, Wanchao Chen, Wen Li, Yan Yang

**Affiliations:** 1 Institute of Edible Fungi, Shanghai Academy of Agricultural Sciences, Key Laboratory of Edible Fungi Resources and Utilization (South), Ministry of Agriculture, National Engineering Research Center of Edible Fungi, Shanghai 201403, China; liupeng@saas.sh.cn (P.L.); 18362436912@163.com (Y.W.); lizhengpeng@saas.sh.cn (Z.L.); wudi@saas.sh.cn (D.W.); zhangzhong@saas.sh.cn (Z.Z.); chenwanchao@saas.sh.cn (W.C.); liwen@saas.sh.cn (W.L.); 2 Shanghai Engineering Research Center of Molecular Therapeutics and New Drug Development, School of Chemistry and Molecular Engineering, East China Normal University, Shanghai 200062, China; 52280946004@stu.ecnu.edu.cn

**Keywords:** *Sanghuangporous vaninii*, polyphenols, antioxidant activity, isolation, network pharmacology

## Abstract

*Sanghuangporous vaninii*, as an edible and medicinal macrofungus, represents a high source of polyphenols with considerable antioxidant activities. However, due to the significant differences in polyphenol content and bioactivity caused by different cultivation substrates, its antioxidant mechanism has not been fully determined. In this paper, five groups of *S. vaninii* fruiting bodies were collected from cultivation substrates from different areas. The ethanol extracts of mulberry sawdust from Haining City (HNMS) had the highest polyphenol content, as well as excellent antioxidant activity. HNMS3, a polyphenol component with promising antioxidant capacity, was further isolated through optimization with different extractants, silica gel column chromatography, and thin layer chromatography analysis. UPLC-Q-TOF-MS analysis showed that HNMS3 was composed of 33 compounds, corresponding to 257 targets of oxidative stress by network pharmacology analysis, which were strongly associated with mental health and neurodegenerative diseases. Protein–protein interaction and molecular docking analysis indicated that eight hub genes (PPARG, IL-6, STAT3, PTGS2, SRC, MTOR, ERS1, and EGFR) are attributed to the regulation of the key compounds hispidin, inoscavin A, inoscavin_C, and phellibaumin B. Consequently, this study obtains *S. vaninii* polyphenolic component HNMS3 with excellent antioxidant capacity, simultaneously revealing its potential antioxidant mechanisms, providing new insights into the application of *S. vaninii*.

## 1. Introduction

Medicinal fungi possess high nutritional value and great potential, with multiple biological activities in both in vitro and in vivo settings, which have emerged as a prominent focus in the healthcare, pharmaceutical, and cosmetic industries [1]. *Sanghuangporous vaninii*, as a typical medicinal fungus, could already be artificially cultivated in the bag and cut log and has been applied for the treatment of diarrhea, hemorrhage, cancer, and menstrual-related disorders [2,3]. The exertion of these activities is mainly attributed to the presence of various active components in the fruiting body of *S. vaninii*, including polyphenols, flavonoids, polysaccharides, etc. Among them, polyphenols are the main active components of *S. vaninii* with a wide range of health-promoting effects. Recently, studies revealed convincing evidence that *S. vaninii* polyphenols exhibited strong radical scavenging, anti-lipid peroxidation, anti-DNA damage capacity, and antiproliferative activity against cancer cells [4]. Especially, polyphenols from *S. vaninii* harbored a high antioxidant capacity, which might be related to its rich chemical components, such as hispidin, hispolon, caffeic acid, etc., considered as important candidates for functional food and medicine [4,5]. However, most research is focused on the biological activity of crude polyphenols from *S. vaninii*, which has become a bottleneck in studying their mechanism of action and further applications.

Due to the significant differences in the quality of cultivation substrates in different regions, it can lead to variations in the composition of fruiting bodies, thereby affecting their bioactivity. It has been proved that the active ingredients in *Ganoderma lucidum* fruiting bodies cultivated on different substrates were altered, including the content of triterpenoids, sterols, and polysaccharides [6]. Additionally, the cultivation of the same mushroom strains in different regions under the same environmental conditions can significantly alter its chemical composition. Vieira Junior WG et al. [7] found that the fungus *Agaricus subrufescens* grown in China, USA, Brazil, Taiwan, and Japan, among others, exhibited significantly different levels of ergosterol and vitamin D2. The occurrence of this phenomenon may be related to the differences in the quality of its cultivation substrate. Since the 1990s, the *Sanghuangporus* genus has been artificially domesticated in China. Currently, it is widely planted in different regions of China, mainly in Zhejiang, Shandong, Jilin, Hubei, Shanxi, and Heilongjiang Provinces, etc., and grows on sawdust cultivation substrates of mulberry, poplar, oak, jujube, and walnut trees. An important prerequisite for the further application and polyphenol extraction is to screen high polyphenol content of *S. vaninii*. Nevertheless, the comparative study of components (especially the evaluation of polyphenol content) in *S. vaninii* fruiting bodies from different cultivation substrates or regions remains a mystery.

Accordingly, five groups of *S. vaninii* fruiting bodies from different sources were collected in this study. The antioxidant capacity and polyphenol content of alcohol extracts from the *S. vaninii* fruiting bodies were compared and analyzed, and polyphenol compounds were further separated and enriched by extraction chromatography. Moreover, the components of the sample were analyzed by UPLC-Q-TOF-MS, and a network pharmacology-based strategy was employed for revealing the antioxidant mechanism. The present study aimed to determine the antioxidant mechanisms of five groups of *S. vaninii* while providing a theoretical basis for the separation of polyphenols from *S. vaninii* and opening novel perspectives for its application either in functional food or medicine.

## 2. Materials and Methods

### 2.1. Materials

The cultivated fruiting bodies of *S. vaninii* with different substrates were collected from Haining City (Zhejiang Province), Yulin City (Shanxi Province), and Yichang City (Hubei Province). Vitamin C, 2,2−diphenyl−1−picrylhydrazyl (DPPH), and 2,2′−azinobis (3−ethylbenzothiazoline 6-sulfonate; ABTS) were obtained from Shanghai Yuanye Bio-Technology Co., Ltd. (Shanghai, China). Silicone (200–300 mesh) was obtained from Shanghai Titan Scientific Co., Ltd. (Shanghai, China). Thin-layer chromatography on a silica−gel plate was purchased from Qingdao Bangkai Hi-tech Materials Co., Ltd. (Qingdao, China).

### 2.2. Preparation of Alcohol Extracts from Different Sources of S. vaninii

**Figure 1:**
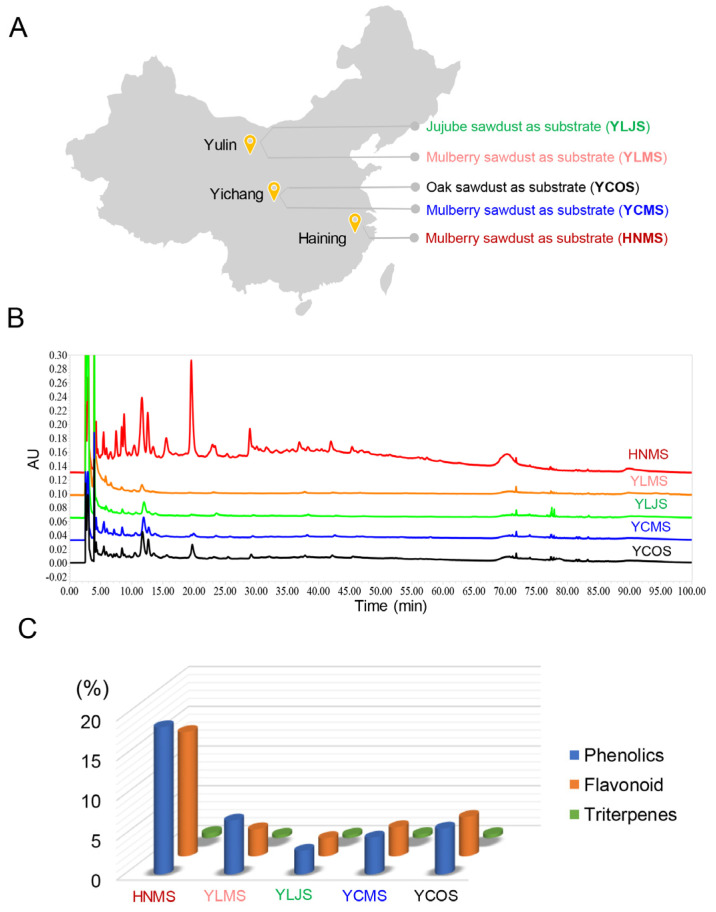
*S. vaninii* fruiting bodies from different regions or cultivation substrates exhibited different composition components. (**A**) Five groups of *S. vaninii* fruiting bodies were collected in China. (**B**) HPLC fingerprint of the ethanol extracts from these five groups of *S. vaninii* fruiting bodies. (**C**) Analysis of key component contents, the values are presented as means (*n* = 3).

*S. vaninii* fruiting bodies were, respectively, obtained and cultivated using sawdust from mulberry in Haining City (HNMS), Yichang City (YCMS), and Yulin City (YLMS); oak in Yichang City (YCOS); and jujube in Yulin City (YLJS) (Figure 1A). *S. vaninii* was cultivated in the cities where sawdust was produced, and the cultivation material was mainly composed of 90% sawdust and 10% bran.

Preparation of alcohol extract: Fruiting bodies of *S. vaninii* were dried, crushed, and sifted to obtain the powder. The crude extracts were carried out twice by mixing 200 g of powder with 3.8 L of 60% ethanol and ultrasound (Ningbo Scientz Biotechnology Co., Ltd., Ningbo, China) at room temperature for 2 h with a power density of 600 W. Subsequently, the sample was centrifuged (10,000× *g*, 20 min) at room temperature, and the supernatant was collected. The supernatant was evaporated by rotary evaporator (BC−R2001, Shanghai Biokai Biochemical Equipment Co., Ltd., Shanghai, China) to remove ethanol and then freeze-dried using a freeze dryer (CHRIST ALPHA 2-4 LD plus, Osterode, Germany) to obtain the ethanol crude extract.

### 2.3. Purification of S. vaninii Polyphenols

60 g of ethanol extract of *S. vaninii* fruiting bodies dissolved in 1.14 L of 20% ethanol solution; the solution was extracted sequentially by equal volumes of petroleum ether, chloroform, ethyl acetate, and N-butanol. Then extracts (named after the last extraction solvent used) were extracted and separated through collecting each organic phase under reduced pressure.

Silica gel column chromatography was applied to purify polyphenols from extracts of *S. vaninii* [8]. Silica gel particles (200–300 mesh) were dried at 105 °C for 30 min and then packed using a dry column method. A total of 0.4 g crude polyphenols were dissolved in 5 mL methanol and laid flat on the surface of silicone gel. The column was washed with 600 mL of eluent (toluene: ethyl acetate: formic acid = 8:20:0.5) at a rate of 2 mL/min, and the eluate was gathered. After vaporizing the eluate, the remainder was dissolved in methanol (1 mg/mL), and then the samples were separated through the thin-layer chromatography method [9]. Plates were then developed twice in a pre-saturated twin-through chamber with toluene: ethyl acetate: formic acid (8:20:1, *v*/*v*/*v*). Visualization of the thin-layer chromatography plates was carried out under UV at 254 nm. Peel off the separated strip with a scraper and dissolve them in methanol. After vaporizing the methanol, the *S. vaninii* polyphenols were obtained.

### 2.4. Determination of Total Polyphenol, Flavonoids, and Triterpenoids Content

A 100 mg sample was dissolved in 25 mL of 60% ethanol, and then the total polyphenol analysis of the sample was determined using the Folin-Ciocalteu colorimetric method as described by Wu M et al. [10] with slight modifications. Folin-Ciocalteu reagent (500 μL) was added to the gallic acid solution, mixed well for 5 min, and then the first 4.5 mL of 7.5% Na_2_CO_3_ was added to the distilled water and incubated at room temperature for 60 min. Absorbance was measured at 740 nm on a 8453 UV−VIS spectrophotometer (Agilent, Palo Alto, CA, USA).

The flavonoids content was detected following the procedure described by Li JM et al. [11] with slight modifications. Take 1 mL methanol extract, prepare 5 mL with 30% methanol, add 0.3 mL 5% sodium nitrite, then add 0.3 mL 10% aluminum nitrate. The mixture was allowed to stand for 60 min at room temperature; 2 mL of 1.0 mol/L sodium hydroxide was added, and the absorbance of the reaction mixture was measured at 485 nm.

Triterpenoids content was referred to Lei et al. [12] with slight modifications. The 200 μL sample solution was placed in a 10 mL volumetric bottle, heated in a water bath until evaporating, mixed with 1 mL of 5% (*w*/*v*) vanillin–acetic acid solution and 1.8 mL of sulfuric acid, and incubated at 70 °C for 30 min. The mixture was then cooled and diluted with acetic acid to 10 mL. Measure the absorbance of the blank at 573 nm using a spectrophotometer. The content was determined using a standard ursolic acid calibration curve. The blank group did not contain a sample solution.

### 2.5. HPLC Analysis

1 mg samples were dissolved in 1 mL methanol. The samples were analyzed by high-performance liquid chromatography (HPLC). The HPLC system consisted of an Alliance water 2695 with an Absorbance UV detector and was equipped with a YMC-PACK ODS-AQ column (YMC, Kyoto, Japan). The flow rate was 1.0 mL/min, the column temperature was maintained at 25 °C, the injection volume was 10 μL, and the UV detection wavelengths were at 254 nm. A gradient elution of acetonitrile (A) and 0.01% acetic acid aqueous solution (B) was adopted; the elution procedure was 0 min, 25% A; 5 min, 25% A; 25 min, 30% A; 60 min, 40% A; 70 min, 95% A; 90 min, 25% A; 100 min, 25% A.

### 2.6. UPLC/Q-TOF MS Analysis

UPLC was performed using a BEH C18 (100 × 2.1 mm, 1.7 µm) (Waters, Milford, MA, USA). The buffer system composed of mobile phase A (water) and mobile phase B (acetonitrile) compositions was separated using the following gradient elution: 0~2 min, 95% A; 2~50 min, 0% A; 55 min, 0% A; 55.1 min, 95% A; 60 min, 95% A. The velocity of flow mobile phase was 0.3 mL/min, and the column temperature was 40 °C. The mass spectrometer was conducted with negative electrospray ionization (ESI), and MS scans were run over a range of *m*/*z* 50 to 1500. The detailed conditions are as follows: capillary voltage 2.8 kV, source block temperature 120 °C, desolvation temperature 300 °C, desolvation gas flow 600 L/h, and cone gas flow 50 L/h.

### 2.7. Determination of Antioxidant Activity

The antioxidant activities of samples were evaluated by the DPPH assay and ABTS assay, which were widely used to detect the antioxidant activity. Detailed experimental methods as described by Rumpf J et al. [13]. The percentage inhibition was calculated using the formula:

% inhibition = (Dc − Dt) × 100/Dc

Dc is the absorbance of blank (excluding the extract), and Dt is the absorbance of the sample with the extract.

### 2.8. Network Pharmacology Analysis

#### 2.8.1. Determining of Gene Targets

The Traditional Chinese Medicine Systems Pharmacology Database and Analysis Platform (TCMSP) was employed to screen bioactive components, and then the effective components were determined by applying oral bioavailability (OB, OB ≥ 30%), drug likeness (DL, DL ≥ 0.18), and Barrier Permeability (BBB) ≥ −0.30 [14]. All gene associations for these bioactive components were collected from the Swiss TargetPrediction and GeneCards databases [15]. The keywords ‘oxidative stress’ were, respectively, entered into the GeneCards and Drugbank databases for searching all target genes. The Uniprot ID of all target genes was normalized in the Uniprot database.

#### 2.8.2. Network Construction

STRING database was applied to screen the interactions among a group of PSV-liver injury-ferroptosis shared targets. Next, the protein–protein interaction (PPI) network and integrated network of component-target-pathway were visualized by Cytoscape (Version 3.9.1) software.

#### 2.8.3. Molecular Docking

The molecular docking method can refer to previous study [16]. The 3D structure of the small molecule ligand was obtained through the PubChem database and saved as an SDF file. Then the RCSB Protein Data Bank was used to screen the crystal structures of protein targets with high resolution as molecular docking receptors. The Molecular Operating Environment 2015.10 software was used to minimize the energy of the compounds, pretreat the target proteins, and search for active pockets. Finally, MOE 2015 was run for molecular docking, and the number of operations was set to 50 times. The binding activity of the two was evaluated according to the binding energy, and the results were visualized by PyMOL2.6.0 and Discovery studio2019 software.

### 2.9. Statistical Analysis

The results were analyzed by a one-way analysis of variance (ANOVA) and two-way ANOVA by Tukey’s post hoc test. Normality and homogeneity of the results were evaluated by the Shapiro–Wilk test. *p* values less than 0.05 were considered to be significant.

## 3. Results and Discussion

### 3.1. Polyphenolic Content and Antioxidant Activity of S. vaninii from Different Regions

#### 3.1.1. The Content of Polyphenols 

The cultivation substrates in different regions are the key factors leading to differences in the composition of *S. vaninii* fruiting bodies, which in turn result in significant variations in their activity. In this study, five samples of *S. vaninii* fruiting bodies from different regions (cultivation substrates) were collected (Figure 1A), and the key components in their ethanol extracts were determined. The HPLC fingerprint showed that secondary metabolites with high polarity, such as polyphenols, were present in the fruiting bodies cultivated with different substrates, while the alcohol extract of HNMS exhibited more peaks, and compounds with lower polarity were more abundant compared to other samples (Figure 1B). The contents of polyphenols, flavonoids, and triterpenes were analyzed; it consistently illustrated that the polyphenols content in all samples was significantly higher than that of flavonoids and triterpenes. Furthermore, the sample of HNMS exerted the highest polyphenols content at (16.09 ± 0.06) %, and the flavonoids and triterpenes content of it were also higher than that of YCOS, YCMS, YLJS, and YLMS (Figure 1C). Increasing studies suggested that the major components in *S. vaninii* consist of polyphenols, triterpenoids, and flavonoids, which are also key compounds for its health-improving effects, such as pulmonary protection, hypoglycemic properties, sleep improvement, gout mitigation, etc. [17]. Among them, polyphenols components play the most crucial role in their biological activity. It was reported that polyphenols components in these medicinal fungi exerted excellent anti-carcinogenesis, anti-inflammatory, anti-obesity, and anti-oxidant activities [18]. In summary, compared to other cultivation substrates in different regions, *S. vaninii* HNMS possessed abundant polyphenol content, which is the cornerstone of its excellent biological activity.

#### 3.1.2. The Antioxidant Capacity

**Figure 2:**
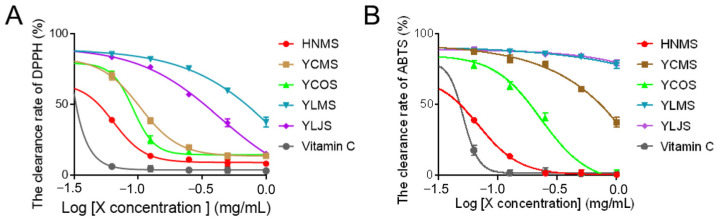
Analysis of antioxidant capacity in vitro of alcohol extracts from *S. vaninii* fruiting bodies. (**A**) DPPH radical scavenging assay and (**B**) ABST radical scavenging assay were employed. All the values are presented as means ± standard error of mean (SEM) (*n* = 3).

Oxidative stress has been implicated as a causative factor in many diseases, which is defined as the imbalance between the production of reactive oxygen species and the endogenous antioxidant defence system [19]. ABTS•+ radical cation-based assays are among the most abundant antioxidant capacity assays, together with the DPPH radical −based assays, which are recommended methods to determine the antioxidant activity [20]. Accordingly, the antioxidant activity of samples was detected employing ABTS and DPPH assays in this study. The data revealed that HNMS showed obvious free radical scavenging capacity against DPPH and ABTS, which is better than other samples; especially, 1 mg/mL of HNMS can achieve a clearance rate of 92% and 99.5% for DPPH and ABTS, respectively (Figure 2). Due to the high content of polyphenols in HNMS, YCOS, and YCMS, as well as their excellent antioxidant capacity, indicating that polyphenols might be the main antioxidant active substances in *S. vaninii*. Gao H et al. [4] indicated that phenolic-rich extracts from *S. vaninii* as a valuable source of natural antioxidative ingredients exhibited strong radical scavenging capacity. By comparing the antioxidant activity of flavonoids, polyphenols, and polysaccharides in *Inonotus sanghuang*, Tian XM et al. found its free-radical scavenging activity was mainly contributed to by polyphenols [21]. Therefore, *S. vaninii* cultivated with mulberry sawdust as a substrate in Haining City (HNMS) was rich in polyphenols and exhibited the greatest potential as antioxidants for development.

### 3.2. Separation and Purification of Polyphenols from HNMS

#### 3.2.1. Liquid–Liquid Extraction

**Figure 3:**
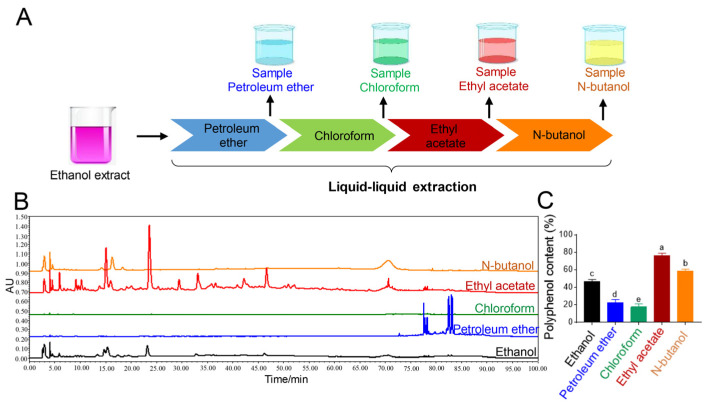
Optimization of extractants. (**A**) Schematic diagram of liquid–liquid extraction. (**B**) HPLC fingerprint and (**C**) polyphenol content of extracts from different extractants. All the values are presented as means ± standard error of mean (SEM) (*n* = 3). Different letters above the bars represent results that were significantly different, *p* < 0.05.

The further separation and purification of polyphenols from HNMS is the key to a deeper understanding of its antioxidant activity and applications. At present, ethanol or methanol is mostly used as an extractant for the extraction of polyphenols, which results in obtaining crude polyphenol extracts [22,23]. However, it is not enough to clarify the key components that exert biological activity. Therefore, the ethanol extract HNMS was further extracted by petroleum ether, chloroform, ethyl acetate, and n-butanol sequentially for separation and enrichment of polyphenol components (Figure 3A). Consequently, the HPLC fingerprint showed that petroleum ether extracts contained components of the lowest polarity that appeared first on the chromatogram, chloroform extracts possessed the least number of compounds, n-butanol extract contained a relatively abundant amount of highly polar components, while ethyl acetate extract contained the largest number of different components (Figure 3B). The polyphenol content was further determined, it confirmed that the extract with ethyl acetate possessed the highest content of polyphenols (74.5%), followed by n-butanol extract (60.02%), which were both significantly higher than that of ethanol extract (Figure 3C). Therefore, further treatment of the alcohol extract with ethyl acetate would be a promising way to obtain polyphenol components from HNMS. The existing report has also coincided with our conclusion; for example, an ethyl acetate extract of a plant had the highest concentrations of phenols compared to the chloroform, n-hexane, n-butanol, and water extracts [24]. Moreover, Deghima A et al. [25] found that ethyl acetate extract showed the highest amounts of phenolic compounds, as well as strong DPPH• radical scavenging ability.

#### 3.2.2. Silica Gel Column Chromatography and Thin Layer Chromatography Analysis

**Figure 4:**
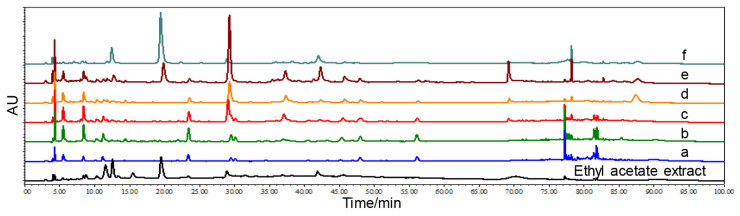
Isolation and purification of HNMS purified by liquid-liquid extraction were further implemented. HPLC fingerprint of ethyl acetate extract and six recombinant samples obtained by silica gel column chromatography analysis.

The ethyl acetate extract was further separated and enriched through silica gel column chromatography, which is widely used for the isolation of polyphenols [26,27]. Tentative identification was carried out using thin-layer chromatography, followed by subsequent merging of similar samples. In this study, a total of 135 samples were collected, which were visualized under UV light irradiation. Merging sample-based profiling data were implemented, resulting in six samples (a~f). The six merged samples were presented by HPLC (Figure 4). Compared with the ethyl acetate extract, samples a~f exhibited significantly different fingerprint spectra, suggesting that the acetate extract of HNMS was effectively separated, resulting in the presentation of new compound compositions. To further purify and separate the polyphenol components (samples a~f) enriched by the above-mentioned silica gel column chromatography method, thin-layer chromatography analysis was employed with toluene, ethyl acetate, and formic acid as the developing agents. The data displayed that samples a~f were separated into 29 new samples, among which sample c was separated into six new samples and sample d was exhibited in seven samples. Further HPLC analysis was performed on these 29 samples, and then sample merging was carried out to obtain 5 recombinant samples (HNMS1~HNMS5).

#### 3.2.3. The Antioxidant Activity of Different Polyphenol Components from HNMS

**Figure 5:**
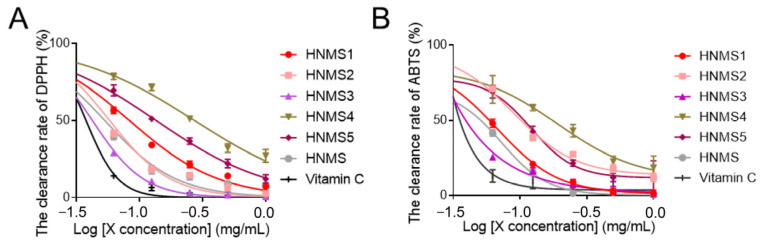
Analysis of antioxidant capacity in vitro of HNMS1 ~HNMS5. (**A**) DPPH radical scavenging assay and (**B**) ABST radical scavenging assay were employed. All the values are presented as means ± standard error of mean (SEM) (*n* = 3).

The antioxidant capacity of HNMS1~HNMS5 was further evaluated and compared. It illustrated that HNMS3 demonstrated significantly higher DPPH and ABTS radical scavenging activities than the other four samples (Figure 5A,B) and showed the highest antioxidant potency. Activities results were compared with the HNMS for DPPH and ABTS radical scavenging activities; the antioxidant scavenging activities of HNMS3 exhibited higher activity. The clearance rate of DPPH by HNMS3 (0.03125~1 mg/mL) ranged between 33.5% and 98.89%, and its clearance rate for ABTS was between 31.7% and 99.25%, showing a dose-dependent manner. The HPLC fingerprint of HNMS3 is shown in Figure S1. Concludingly, HNMS was ultimately separated and enriched into five components (HNMS1~HNMS5), among which HNMS3 exerted the highest potential for antioxidant activity, implying that it might be a potential substance for the development of antioxidant function.

### 3.3. Network Pharmacology Analysis

#### 3.3.1. The Effective Active Compounds in HNMS3 and Key Target Genes

**Figure 6:**
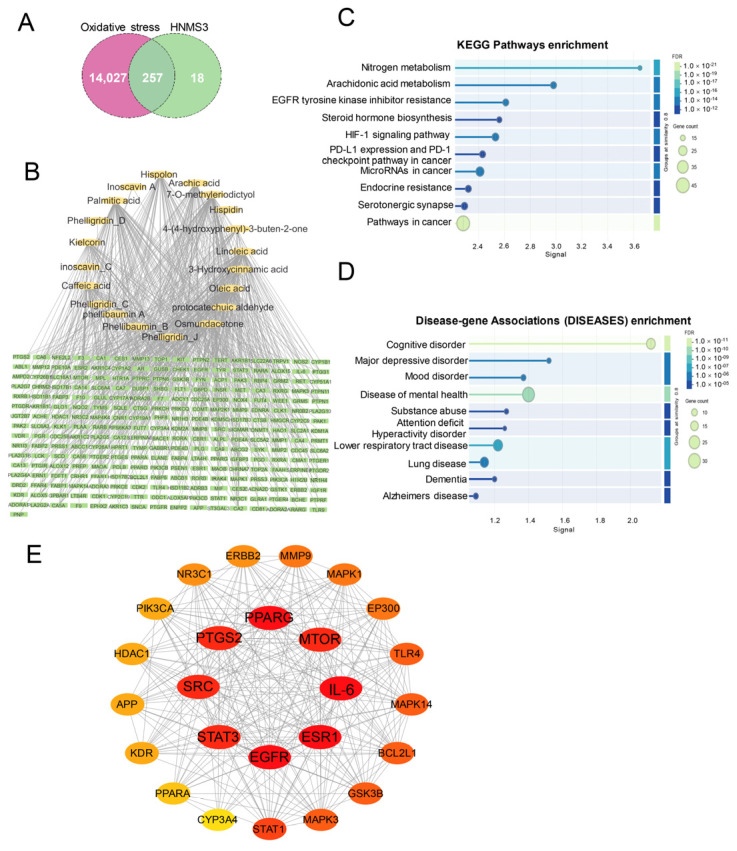
Network pharmacology analysis. (**A**) Venn diagram of the targets of the active ingredients and oxidative stress-related targets. (**B**) The network of the 257 common targets and 20 compounds from HNMS3. (**C**) KEGG and (**D**) Disease–gene Associations (DISEASES) enrichment analysis of the common targets. (**E**) PPI network of the common and core targets, the deepened node color was proportional to the degree of interaction.

To further explore the mechanism of antioxidant activity of HNMS3, the components of HNMS3 were further analyzed by UPLC-Q-TOF-MS, which is a feasible and effective method to screen and identify components from extract [28,29]. Combining the Chemical Spider, PubChem, and Lipidomics Gateway databases, 33 compounds were identified in HNMS3 (Figure S2, Table S1). Twenty effective active compounds were screened from HNMS3 based on OB, DL, and BBB databases (Figure 6B). These compounds were imported into the Swiss TargetPrediction and GeneCards databases, and then a total of 275 potential targets of HNMS3 active ingredients were obtained. The potential oxidative stress targets were 14,284. The Venn diagram showed that the intersection of active compound targets of HNMS3 and targets of oxidative stress included 257 common targets (Figure 6A). The interaction diagram between 257 common targets and 20 key bioactive compounds in the HNMS3 was shown in Figure 6B. It emphasized that osmundacetone, hispolon, hispidin, and caffeic acid played a crucial effect in the antioxidant activity of HNMS3, which has been proven to act as an antioxidant that has been shown to possess multiple bioactivities, including neuroprotective and immunomodulatory effects, etc. [30,31,32,33]. Among them, hispolon and hispidin have been identified as one of the most important compounds from the genus *Sanghuangporus* [34,35]. The KEGG pathway enrichment analysis of the 257 core targets mainly involved in nitrogen metabolism, arachidonic acid metabolism, EGFR tyrosine kinase inhibitor resistance, steroid hormone biosynthesis, and HIF-1 signaling pathway, etc. (Figure 6C). Previous research revealed that there was a close relationship between the disruption of these pathways and the antioxidant system disruption [36,37,38,39,40]. Interestingly, Disease-gene Associations (DISEASES) enrichment analysis showed that the core targets affected by HNMS3 treatment mainly involved cognitive disorder, major depressive disorder, mood disorder, etc. (Figure 6D). This data highlighted the importance of HNMS3 in regulating mental health and neurodegenerative diseases. There are a large number of studies indicating that polyphenol-rich components derived from natural products could improve cognitive function and exhibit brain-health-promoting effects [41]. Furthermore, the key compounds osmundacetone, hispolon, hispidin, and caffeic acid have been proved to possess excellent neuroprotection through resisting oxidative stress response [30,33,42,43]. This conclusion coincides with the above analysis results. Therefore, the results provided a new perspective for the biological activity and application research of *S. vaninii*.

#### 3.3.2. Interaction Network of Key Targets and Molecular Docking

**Figure 7:**
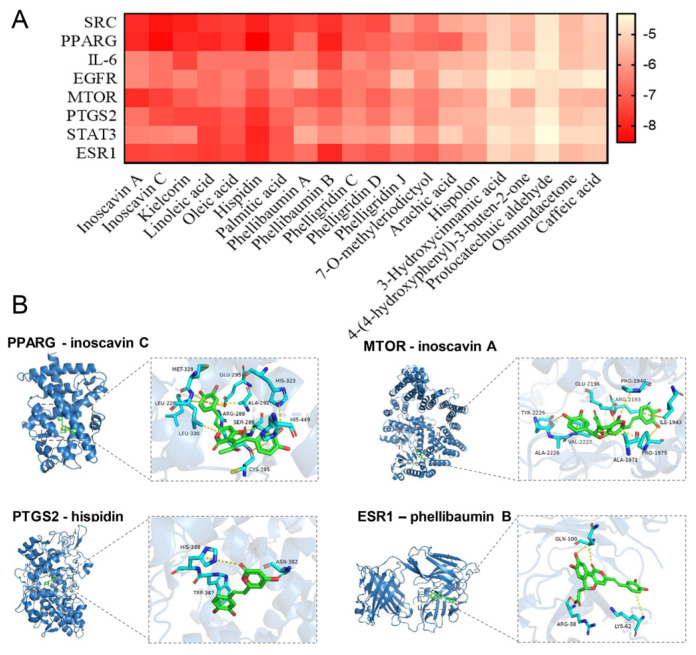
Molecular docking. (**A**) Molecular docking analysis, the deepened node color of active compounds was proportional to the degree of interaction. The heat map of binding energy (kcal/mol) of key targets and compounds of HNMS3. (**B**) Visualized display of molecular docking between representative active compounds and core targets.

These common targets were imported to the STRING database for construction of a PPI network; peroxisome proliferator-activated receptor gamma (PPARG), mechanistic target of rapamycin (MTOR), interleukin 6 (IL-6), estrogen-receptor 1 (ESR1), epidermal growth factor receptor (EGFR), signal transducer and activator of transcription 3 (STAT3), non-receptor tyrosine kinase (SRC), and prostaglandin-endoperoxide synthase 2 (PTGS2) were identified as core targets by Cytoscape (Figure 6E). Previous reports revealed that PPARG, IL-6, STAT3, MTOR, and PTGS2, as cross cores, participated in oxidative stress response [44,45,46,47]. For example, PTGS2 is a biomarker of ferroptosis, a form of regulated cell death caused by the accumulation of iron-mediated lipid peroxidation, which was reported to be inhibited by flavonoids [48]. Furthermore, a polyphenol from an edible and medicinal plant has been proved to possess antioxidant activity, which was attributed to regulating the expressions of PTGS2 and ESR1, etc., involving the cancer signaling pathway and estrogen signaling pathway [49]. This basically coincides with our conclusion. To assess interaction between screened active compounds and hub targets, molecular docking was applied to validate the findings from network pharmacology. The results showed that the binding energies of key active compounds of HNMS3 to the core targets were all less than −4.4 Kj/mol, suggesting that they could bind spontaneously to these core targets (Figure 7A). Especially hispidin, inoscavin A, inoscavin C, and phellibaumin B stably band to these hub targets, especially with a specific binding to PPARG, MTOR, PTGS2, and ERS1, which were visually displayed in Figure 7B. Hispidin, inoscavin A, phellibaumin B, and inoscavin C are natural products with significant antioxidant activities that are widely found in various kinds of *Sanghuangporus* spp., which are potential medicinal substances in *Sanghuangporus* [50,51,52]. Accordingly, it revealed that HNMS3 exerted its antioxidant capacity with multi-target, multi-pathway through binding to core targets, which would deepen and enrich the understanding of the potential mechanism of HNMS3 from *S. vaninii* against oxidative stress.

## 4. Conclusions

HNMS rich in polyphenols was separated from the fruiting bodies of *S. vaninii* cultivated in mulberry sawdust at Haining City. HNMS3 with excellent antioxidant capacity was further isolated and purified from HNMS. HNMS3 is composed of 33 compounds, which were identified by UPLC-Q-TOF-MS, among which 20 active ingredients were found, corresponding to 257 intersection targets of oxidative stress. The PPI network showed that the key proteins involved were PPARG, MTOR, IL-6, ESR1, EGFR, STAT3, SRC, and PTGS2, which might be attributed to the regulation of the key compounds hispidin, inoscavin A, inoscavin C, and phellibaumin B. Moreover, the core targets affected by HNMS3 treatment mainly involved mental health and neurodegenerative diseases. The molecular docking results indicated key binding activity between PTGS2-hispidin, PPARG-inoscavin C, MTOR-inoscavin A, and ESR1-phellibaumin B. However, additional studies are urgently needed to confirm the significance of the core targets and the core signaling pathway in the antioxidant activity of HNMS3 in the future. Summary: HNMS3 can exert antioxidant capacity by regulating multiple targets and signaling pathways, which would pave the way for the utilization of *S. vaninii* as a promising antioxidant natural resource.

## Data Availability

Data will be made available on request.

## Supplementary Materials

The following supporting information can be downloaded at: https://www.mdpi.com/article/10.3390/antiox13121487/s1. Table S1. Identification of compounds from HNMS3 by UPLC-QTOF-MS. Figure S1. HPLC fingerprint of HNMS3. Figure S2. Component identification of HNMS3 was analyzed by UPLC-Q-TOF-MS. Compounds c1~c33 were 4-(4-hydroxyphenyl)-3-buten-2-one, Osmundacetone, 7-Acetoxycoumarin-3-carboxylic acid, Hispidin, Citrinin, Phelligridin_J, 7-O-methyleriodictyol, Palmitic acid, Linoleic acid, Oleic acid, phellibaumin A, Phelligridin_C, Phelligridin_D, inoscavin_D, Phellibaumin_B, Hydroxy-docosanoic acid, inoscavin_C, interfungin_B, Kielcorin, Hydroxy-tetracosanoic acid, Inoscavin A, Davallialactone, Hypholomine B, Hypholomine A, SCHEMBL8676491, Pinillidine, Phelligridin_H, Phelligridin_I, Phelligridin_I_500, SCHEMBL8859595, Phelligridimer A, Protocatechuic aldehyde and Caffeic acid, respectively.
