# Supplementary material for: Isolation and Antioxidant Mechanism of Polyphenols from Sanghuangporous vaninii
Source: Antioxidants (Basel). 2024 Dec 5;13(12):1487. doi: 10.3390/antiox13121487 (PMC11674009; doi:10.3390/antiox13121487)
Supplement: Supplementary file 1 [file antioxidants-13-01487-s001.zip › antioxidants-3308144-supplementary.pdf]

Table S1. Identification of compounds from HNMS3 by UPLC-QTOF-MS.

No.	Components	Formula	Neutral mass (Da)	Observed RT (min)
1	4-(4-hydroxyphenyl)-3-buten-2-one	$C_{10}H_{10}O_2$	162.06808	19.58
2	Osmundacetone	$C_{10}H_{10}O_3$	178.06299	16.73
3	7-Acetoxycoumarin-3-carboxylic acid	$C_{12}H_8O_6$	248.03209	24.19
4	Hispidin	$C_{13}H_{10}O_5$	246.05282	18.96
5	Citrinin	$C_{13}H_{14}O_5$	250.08412	21.69
6	Phelligridin_J	$C_{13}H_6O_8$	290.00627	76.19
7	7-O-methyleriodictyol	$C_{16}H_{14}O_6$	302.07904	19.6
8	Palmitic acid	$C_{16}H_{32}O_2$	256.24023	59.4
9	Linoleic acid	$C_{18}H_{32}O_2$	280.24023	47.8
10	Oleic acid	$C_{18}H_{34}O_2$	282.25588	63.17
11	phellibaumin A	$C_{19}H_{12}O_7$	352.0583	26.19
12	Phelligridin_C	$C_{20}H_{12}O_7$	364.0583	29.17
13	Phelligridin_D	$C_{20}H_{12}O_8$	380.05322	26.45
14	inoscavin_D	$C_{21}H_{16}O_8$	396.08452	28.68
15	Phellibaumin_B	$C_{22}H_{16}O_9$	424.07943	25.51
16	Hydroxy-docosanoic acid	$C_{22}H_{44}O_3$	356.32905	68.61
17	inoscavin_C	$C_{23}H_{16}O_8$	420.08452	28.6
18	interfungin_B	$C_{23}H_{20}O_8$	424.11582	14.44
19	Kielcorin	$C_{24}H_{20}O_8$	436.11582	15.79
20	Hydroxy-tetracosanoic acid	$C_{24}H_{48}O_3$	384.36035	72.5
21	Inoscavin A	$C_{25}H_{18}O_9$	462.09508	25.03
22	Davallialactone	$C_{25}H_{20}O_9$	464.11073	21.05
23	Hypholomine B	$C_{26}H_{18}O_{10}$	490.09	23.24
24	Hypholomine A	$C_{26}H_{18}O_9$	474.09508	25.4
25	SCHEMBL8676491	$C_{27}H_{20}O_{10}$	504.10565	32.61
26	Pinillidine	$C_{28}H_{22}O_{10}$	518.1213	30.62
27	Phelligridin_H	$C_{33}H_{18}O_{13}$	622.07474	31.11
28	Phelligridin_I	$C_{33}H_{20}O_{13}$	624.09039	27.3
29	Phelligridin_I_500	$C_{33}H_{20}O_{13}$	624.09039	26.28
30	SCHEMBL8859595	$C_{33}H_{24}O_{12}$	612.12678	27.85
31	phelligridimer A	$C_{52}H_{32}O_{20}$	976.14869	26.76
32	protocatechuic aldehyde	$C_7H_6O_3$	138.03169	11.23
33	Caffeic acid	$C_9H_8O_4$	180.04226	13.56

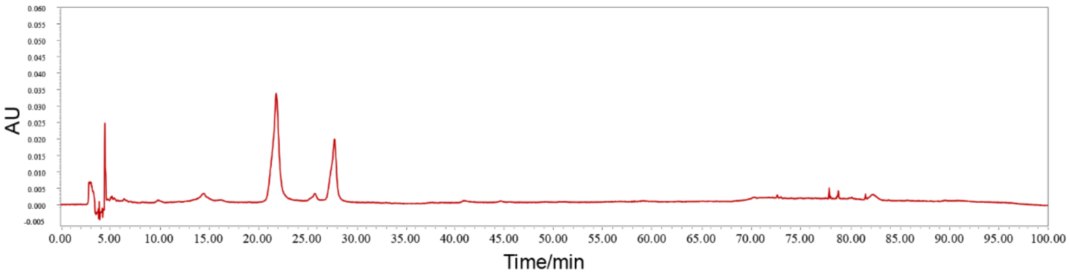

Figure S1. HPLC fingerprint of HNMS3

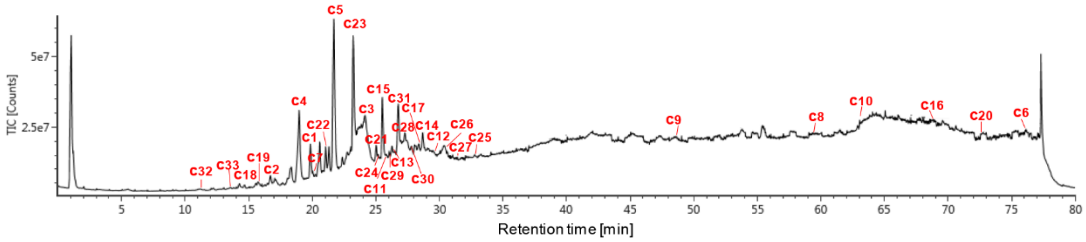

Figure S2. Component identification of HNMS3 was analyzed by UPLC-Q-TOF-MS. Compounds c1~ c33 were 4-(4-hydroxyphenyl)-3-buten-2-one, Osmundacetone, 7-Acetoxycoumarin-3-carboxylic acid, Hispidin, Citrinin, Phelligridin_J, 7-O-methyleriodictyol, Palmitic acid, Linoleic acid, Oleic acid, phellibaumin A, Phelligridin_C, Phelligridin_D, inoscavin_D, Phellibaumin_B, Hydroxy-docosanoic acid, inoscavin_C, interfungin_B, Kielcorin, Hydroxy-tetracosanoic acid, Inoscavin A, Davallialactone, Hypholomine B, Hypholomine A, SCHEMBL8676491, Pinillidine, Phelligridin_H, Phelligridin_I, Phelligridin_I_500, SCHEMBL8859595, Phelligridimer A, Protocatechuic aldehyde and Caffeic acid, respectively.
